# Recent Developments in Vaccines for Bovine Mycoplasmoses Caused by *Mycoplasma bovis* and *Mycoplasma mycoides* subsp. *mycoides*

**DOI:** 10.3390/vaccines9060549

**Published:** 2021-05-24

**Authors:** Katarzyna Dudek, Ewelina Szacawa, Robin A. J. Nicholas

**Affiliations:** 1 Department of Cattle and Sheep Diseases, National Veterinary Research Institute, 24100 Pulawy, Poland; ewelina.szacawa@piwet.pulawy.pl; 2 The Oaks, Nutshell Lane, Farnham, Surrey GU9 0HG, UK; robin.a.j.nicholas@gmail.com

**Keywords:** vaccine, cattle, *Mycoplasma bovis*, *Mycoplasma mycoides* subsp. *mycoides*

## Abstract

Two of the most important diseases of cattle are caused by mycoplasmas. *Mycoplasma bovis* is a world-wide bovine pathogen that can cause pneumonia, mastitis and arthritis. It has now spread to most, if not all, cattle-rearing countries. Due to its increasing resistance to antimicrobial therapy, vaccination is the principal focus of the control of infection, but effective vaccines are currently lacking. Despite being eradicated from most parts of the world, *Mycoplasma mycoides* subsp. *mycoides*, the cause of contagious bovine pleuropneumonia (CBPP), continues to plague sub-Saharan Africa, affecting at least 25 countries. Numerous new experimental vaccines have been developed over the last 20 years to improve on protection afforded by the T1/44, a live vaccine in continuous use in Africa for over 60 years, but none so far have succeeded; indeed, many have exacerbated the disease. Tools for diagnosis and control are adequate for eradication but what is necessary are resources to improve vaccine coverage to levels last seen in the 1970s, when CBPP was restricted to a few countries in Africa. This paper summarizes the results of the main studies in the field of experimental mycoplasma vaccines, reviews data on commercially available bacterin vaccines and addresses issues relating to the search for new candidates for effective vaccines to reduce economic losses in the cattle industry caused by these two mycoplasmas.

## 1. Introduction

Mycoplasmas are the smallest self-replicating bacteria and are pleomorphic, have a low GC content and are devoid of a cell wall [1]. Out of over 200 recognized mycoplasma species, 13 have been identified in cattle, with *Mycoplasma bovis* and *Mycoplasma mycoides* subsp. *mycoides* being the most pathogenic and responsible for significant economic losses [2].

*M. bovis* is the etiological agent of many disorders in cattle with different clinical manifestations, such as pneumonia, mastitis, arthritis, otitis, keratoconjunctivitis, endocarditis and brain disorders [3]. *M. bovis* has the ability to form an adherent biofilm, which facilitates its survival in the host and aids the chronic course of the disease [1,4,5]. It is known that *M. bovis* is able to evade the host immune system most of all due to high antigenic variability of the strains, its intracellular persistence in both phagocytic and non-phagocytic cells and the immune response modulation by the bacteria [6,7,8,9]. Due to the increasing resistance of European field strains to most antimicrobials with the exception, so far, of the fluoroquinolones, and overall difficulties in *M. bovis* therapy, the only principal strategy for control of these infections is the use of effective vaccines [10,11]. Many studies have been done using experimental vaccines but, to date, commercially available vaccines are available only in the United States, and their efficacy is not fully satisfactory [12].

Contagious bovine pleuropneumonia (CBPP), one the great historic plagues of cattle alongside the now eradicated rinderpest, continues to inflict serious losses on livestock in many parts of sub-Saharan Africa [13]. But why is CBPP continuing to cause problems when it has been eradicated from Europe, Australia, Asia and North America? Sadly, because of economic hardships, civil wars and droughts affecting the countries where the disease is endemic and the inability to prevent transboundary movement of livestock, control in Africa seems further away than ever. CBPP is a severe pneumonia of cattle caused by the wall-less bacterium *Mycoplasma mycoides* subspecies *mycoides*. The disease is localized in the lungs, where it causes a highly characteristic “marbling” of the lungs in the acute stages and lesions known as a “sequestra” in the chronic form of the disease [14]. Clinical signs include rapid breathing, fever, nasal discharge, anorexia, cough on exertion and sudden death. Mortality rates can exceed 50% when the disease appears for the first time in herds. The mycoplasma is transmitted by close and repeated contact with aerial and environmental infection playing little or no role in its epidemiology. Consequently, it was recognized very early on that the slaughter of affected and contact animals with strict movement restrictions could effectively control the disease [15]. The difficulty, however, is identifying affected animals quickly enough to prevent the disease spreading because, though the lung may be very severely damaged, clinical signs are often lacking [16]; this was particularly true in outbreaks in European herds where cattle remain housed throughout the year. The disease is more obvious in the nomadic herds of sub-Saharan Africa where animals endure a much more hostile environment, leading to higher morbidity and mortality rates than in European cattle.

## 2. *Mycoplasma bovis* Vaccines

### 2.1. Experimental Vaccines

The most important information about experimental *M. bovis* vaccines is summarized in Table 1.

#### 2.1.1. Vaccine Design: Types of Vaccines; Vaccination Route; and Protection Level

Inactivated vaccines are the most commonly used in studies to prevent infections with *M. bovis*. However, it is generally considered that inactivated vaccines have some disadvantages, including their high production costs resulting from the need to culture large amounts of the antigen, as well as possible modifications of the proteins of the strains during subculture. Encouragingly however, the most recent studies using proteomic analysis showed no changes in the major membrane proteins and protective antigens after continuous passaging of the *M. bovis* strain in vitro [17]. Furthermore, due to the 10-fold greater growth rate of the strain as it is subcultured, the production costs of such inactivated vaccines seem to be significantly reduced.

Protection against an intratracheal challenge with a homologous *M. bovis* strain was observed in calves following a single subcutaneous injection with an experimental bacterin vaccine [8]. This vaccine significantly reduced clinical disease, upper-respiratory tract colonization by the bacterium and lung lesions. The vaccine effectively stimulated anti-*M. bovis* antibodies which remained higher than the controls until the end of the study 39 weeks post vaccination. The vaccine was also effective in stimulating the mucosal immunity in the vaccinated/challenged calves.

Significant protection against the infection with a heterologous *M. bovis* strain was obtained with a single subcutaneous dose of another experimental bacterin vaccine for *M. bovis* infection [18]. The vaccine significantly reduced total clinical and lung lesion scores in the vaccinated/challenged calves, which was confirmed by histological examination. An additional advantage of this vaccine was protection against clinical arthritis which developed only in some challenged animals. This experimental vaccine did not prevent nasal shedding of *M. bovis*; however, this may have been due to the severe challenge inflicted by a double inoculation of the vaccinated calves with the pathogen [18]. Similar to the findings of Dudek et al. [8], the onset of specific humoral response occurred in the vaccinated/challenged calves two weeks post vaccination [8,18]. The increased *M. bovis* antibody titers persisted until the end of the study on day 42 after vaccination, when it decreased to the same level as the challenged-only animals. The serological response was monitored in the vaccinated-only calves, which persisted for 6 months after vaccination [18]. Unfortunately, the results obtained with this saponized vaccine have not been reproduced commercially.

*M. bovis* strains from China attenuated by multiple in vitro passages were used to produce a novel live vaccine for protection against *M. bovis*-related pneumonia [19]. The vaccine was prepared in two forms of attenuation and administered via the nasal cavity. Significant reduction in the mean clinical scores after triple intratracheal challenge with the homologous *M. bovis* strain was observed in the immunized calves. The vaccine protected the calves from mean weight loss recorded in the challenged-only animals. No typical lung lesions or significantly lower mean histopathological scores were recorded in the vaccinated/challenged calves. Some reduction in the *M. bovis* nasal shedding after challenge in the immunized calves was observed; however, *M. bovis* was isolated at the same rate as the challenged-only animals until the end of the study on day 21. Additionally, *M. bovis* nasal shedding was also demonstrated after immunization, which lasted, depending on the form of the vaccine, up to 7 or 12 days post vaccination. Specific humoral immunity started early, 7 days after vaccination, but its duration lasted just over 30 days post vaccination. However, the vaccine did not fully prevent *M. bovis* dissemination in the host and did not stimulate the mucosal immunity. In the author’s opinion, the less attenuated *M. bovis* strain with the overall assessed protection rate of 81.3% was the most promising candidate for a live *M. bovis* vaccine. This strain seemed to be more immunogenic, although *M. bovis* nasal shedding after the immunization lasted much longer in this case [19].

Using more modern approaches, a sub-unit vaccine was prepared based on previously selected *M. bovis* antigens, such as the *M. bovis* membrane fractions, the whole cell extracts and the nine recombinant proteins, including PdhA, Tuf, PepA, LppB, O256, OppA, DeoB, P81 and PepQ, formulated with the adjuvant combination for effective Th-17 response [20,21]. An elevated humoral response to the *M. bovis* antigens, including serum IGg1 and IGg2 titers, was observed following a single subcutaneous inoculation. However, despite also stimulating cell-mediated responses, indicated by peripheral blood mononuclear cell (PBMC) proliferation and cytokine expression results, including the expected IL-17 response, the vaccine did not protect the calves against *M. bovis* infection. Additionally, PBMC cytokine expression was not completely reflected in the lung cytokine determination. Additionally, the vaccine did not prevent *M. bovis* lung colonization. Some beneficial results of this vaccine were seen, such as weight gain and slightly fewer lung lesions, but the differences noted were not statistically significant. Unfortunately, successful challenge was not fully achieved in this study, despite the inoculation of calves with a suspension of two *M. bovis* strains and challenge being preceded by inoculation with bovine herpesvirus 1 (BHV-1) that causes immunosuppression and should facilitate infection with the target pathogen [20]. One of the reasons for the insufficient *M. bovis* challenge could be too low an infecting dose compared to those used in other *M. bovis* vaccine studies [8,18,22].

The BHV-1/*M. bovis* co-infection model was also used in a trial of a *M. bovis* recombinant-based vaccine aimed at the control of bovine chronic pneumonia and polyarthritis syndrome (CPPS) in feedlot cattle. Despite the general induction of balanced Th1/Th2 responses to some previously selected *M. bovis* antigens, a single subcutaneous dose of this vaccine neither inhibited post-challenge *M. bovis* nasal shedding nor significantly reduced the proportion of specific *M. bovis* lung lesions compared to the control [21,23]. Additionally, the vaccine seemed to exacerbate the disease, resulting in post-challenge deaths and the increased dissemination of the bacteria in the host [23].

To control *M. bovis* infections, vaccines based on a combination of *M. bovis* cell extracts and membrane fractions or membrane fractions alone were prepared [22]. In this study, the same co-infection model was used as before [20]; however, the interval between the BHV-1 and *M. bovis* challenges was shorter [22]. Moreover, a second vaccination was applied 22 days after the first, hopefully increasing the efficacy of this vaccine. Despite satisfactory pre-challenge antibody responses to the antigens and whole cell combination, protection against experimental infection with *M. bovis* was not achieved, though fewer lung lesions were seen with this vaccine. Additionally, the vaccine, regardless of its composition, did not protect against the upper respiratory tract and lung colonization by *M. bovis* and its dissemination in the host. In at least one sample of the diseased lungs from the calves of each group, *Pasteurella* spp. was isolated, which additionally complicates the reliable assessment of the course of the disease [22].

Another attempt to develop an effective sub-unit vaccine against *M. bovis* involved a combination of *M. bovis* glyceraldehyde-3-phosphate (GAPDH) and *M. bovis* cell extracts prepared from the three field isolates [24]. In this case, two vaccinations were also used, but with a 42-day interval. For challenges, the BHV-1/*M. bovis* co-infection model was utilized, but included a suspension of three *M. bovis* isolates. Unfortunately, there was insufficient cell-mediated response to the *M. bovis* recall antigens. A strong humoral immune response was observed based on the IgG1 and IgG2 serum responses, but this gave no protection against the *M. bovis* challenge. Cell-mediated responses were also insufficient. In some cases, even disease exacerbation was observed based on the estimation of lung lesions consistent with *M. bovis* CPPS [24].

#### 2.1.2. Adjuvant/Inactivator Used in the Vaccine Preparation

The use of formalin for mycoplasma cell killing in a vaccine for prevention of *M. bovis*-induced arthritis did not bring the expected results [25,26]. The vaccine did not protect against the effects of articular challenge with the virulent *M. bovis* strain, despite effective stimulation of the humoral response. Post-challenge, the clinical disease and the joint lesions in the vaccinated calves were similar to those observed in the non-vaccinates, which was additionally confirmed by histopathology [25,26]. To some extent, the vaccine partially reduced *M. bovis* joint colonization; however, it did not protect against the *M. bovis* spreading from the inoculated to non-inoculated joints, which was observed in both examined groups of calves [25,26]. Similar poor results were seen with another formalinized vaccine which failed to protect against *M. bovis* arthritis in calves [27]. This vaccine did not protect against the disease in the same way as the live vaccine given intraperitoneally, however, unlike the live one administered subcutaneously [27].

A formalinized vaccine using Freud’s complete adjuvant, aimed at protecting against *M. bovis* experimental mastitis in cows, met with limited success. The vaccine did not protect against infection of the quarters or spreading from the challenged to unchallenged quarters, but shortened the duration of infection compared to the control cows [28].

One of the first attempts to develop a multivalent vaccine for calf respiratory disease containing *M. bovis*, *M. dispar*, respiratory syncytial virus and parainfluenza type 3 virus antigens and formulated with an oil adjuvant or Quil A, a saponin-based adjuvant, gave partial protection to natural challenge. The vaccine developed in 1987 in the UK was not commercialized [29,30].

Quil A was also used to prepare a sub-unit vaccine against *M. bovis* pneumonia, but showed no efficacy [31]. Indeed, the authors claimed it provided a good experimental model for challenge studies as it exacerbated disease. The study of Nicholas et al. was the first to develop and use a saponin as both adjuvant and inactivator for *M. bovis* bacterin vaccine in the experimental animal model [18]. In the other experimental bacterin vaccine an adjuvant complex of saponin and Emulsigen^®^ was also used successfully in protection from *M. bovis* challenge. In this novel dual adjuvanted vaccine, saponin also effectively killed *M. bovis* cells [8]. Another example of the use of saponin in an adjuvant complex in *M. bovis* vaccine was its combination with lysozyme dimer (Lydium-KLP™). This vaccine stimulated *M. bovis* IgG responses which persisted for at least 84 days post vaccination in the vaccinated calves. The IgA response was poorly expressed and only slight stimulation was observed until the end of the study at day 63 post vaccination [32]. This vaccine has also shown its stimulating effect on cell-mediated immunity expressed by generally increased T- and B-cell response in the vaccinated calves [33]. Other data involving this vaccine indicated the activation of an acute phase response post vaccination, which was manifested by upregulation of major bovine acute phase proteins, most seen in the increased SAA concentration. Additionally, as a result of the vaccination, stimulation of IFN-γ concentration with no IL-4 response was also observed [34]. Despite the initial beneficial effects of this vaccine, further confirmation using the calf-infection model is required.

Another attempt to develop saponin-based vaccines involved the combination of saponin with DL-alpha-tocopheryl acetate as an adjuvant [35]. The effect of this combination on the immune response of calves was comprehensively evaluated and the results were compared with those obtained for the above-proposed combination of saponin with Emulsigen^®^ and the vaccine based on saponin alone. Both humoral and cellular immune responses were stimulated post vaccination with the three saponin-based adjuvant formulations, but to a different extent depending on the formulation used. One of the differences between the tested adjuvants for *M. bovis* vaccine concerned the stimulation of responses from different T-cell subsets and the concentrations of various cytokines measured. Predominant stimulation of the humoral response in the form of the strongest production of specific *M. bovis* antibodies as well as cellular response, seen in the most evident B-cell response, confirmed previous assumptions on saponin and Emulsigen^®^ as the best combination of all tested.

A recombinant *M. bovis* vaccine study used mixtures of the two commercial adjuvants, i.e., 60% Montanide™ ISA 61VG and curdlan [20]. Despite the assumptions made regarding the effective stimulation of the IL-17 response, the combination of these adjuvants with the appropriately selected *M. bovis* proteins did not protect the calves against *M. bovis* experimental pneumonia. 

Another sub-unit vaccine containing the same *M. bovis* proteins as above plus the additional recombinant protein P48, adjuvanted with Emulsigen™, IDR peptide 1002 and poly I:C, also did not confer protection against *M. bovis* challenge, and even exacerbated the disease [23]. The vaccine with a combination of *M. bovis* total extracts and membrane fractions or membrane fractions alone was formulated with CpG ODN 2007 and 30% Emulsigen™ adjuvants. However, both forms of vaccine did not fulfill the expected protective effects against an *M. bovis* experimental challenge [22]. The lack of protection against the *M. bovis* challenge has also been demonstrated despite the use of various adjuvants, i.e., 30% Emulsigen™ and CpG2007 for another sub-unit vaccine [24].

#### 2.1.3. Age of Vaccinated Animals 

The optimum age to vaccinate cattle is crucial for *M. bovis* vaccines and is often a barrier against effective protection from infections with this pathogen. The greatest difficulties arises from the development of an effective vaccine for the youngest animals in which specific immunity is frequently blocked by a natural barrier in the form of maternal antibodies [18]. In the study of Dudek et al. [8], 5–6-week-old calves were vaccinated successfully with the *M. bovis* bacterin vaccine. Nicholas et al. [18], using a saponin vaccine, immunized even younger experimental animals, at 3–4 weeks old. Slightly older animals, aged 1 to 4.5 months, were used in the vaccine studies for *M. bovis* arthritis [27]. Even older animals, 6–8 months old, were used in the studies on the sub-unit *M. bovis* vaccines, but no protection was seen against *M. bovis* experimental challenge [20,23,24]. Similar to the previous ones, studies conducted on 5–8-month-old cattle with the use of another sub-unit vaccine did not bring the expected beneficial results [22].

#### 2.1.4. Possible Adverse Reactions

There are few reports of adverse postvaccinal reactions with inactivated *M. bovis* vaccines, but this may depend on the route of vaccine administration. In the study of Dudek et al. [8], following subcutaneous administration of the *M. bovis* bacterin vaccine, slightly increased respiratory/heart rates and nasal discharge were observed in some vaccinated calves. Only one calf had a slight local postvaccinal edema at the vaccination site. All these signs resolved after three weeks post vaccination, while most lasted for much less time. Calves showed only mild adverse effects post vaccination with the *M. bovis* bacterin vaccine, mainly local tissue swelling lasting for several days which additionally confirmed the safety of this vaccine [18]. For the live *M. bovis* vaccine, no specific clinical abnormality based on the clinical score assessments was generally stated. However, there are no detailed data on this over time, as was described for the clinical assessment after the challenge. Compared to the other groups, no significant differences in changes in the rectal temperature and mean daily weight gains were observed in the two immunized groups [19]. For the sub-unit *M. bovis* vaccines, no information is available on pre-challenge clinical score assessment in the vaccinated calves [20,22,23,24]. In older studies, local tissue swelling in the form of edematous plaques was observed at the site of injection of most of the calves post subcutaneous vaccination with the live vaccine for *M. bovis* arthritis, which lasted 9 days. No adverse reactions at the site of administration were seen for the formalinized vaccine. For all the vaccine types, a transient elevation of rectal temperature was recorded. Additionally, one calf which was vaccinated intraperitoneally with the live vaccine became infected [27].

### 2.2. Commercial Vaccines

The most important information about commercially available *M. bovis* vaccines is summarized in Table 2. 

The efficacy of two bacterin *M. bovis* vaccines commercially available in the United States was tested in a blinded, systematically randomized field trial on a total of 200 special-fed veal calves. The first of the analyzed vaccines Mycomune^®^ R (BIOMUNE Co., Lenexa, KS, USA) was generally administered according to the manufacturer’s instruction. Larger deviations from the manufacturer’s instruction were made for the second tested vaccine, Pulmo-Guard™ MpB (American Animal Health, Inc., Grand Prairie, TX, USA), which was administered to calves more than two weeks younger than recommended, and the vaccination interval was also shorter. The only post-vaccination adverse reaction described here was subcutaneous granuloma at the injection site in two animals for Mycomune^®^ R, while no adverse events at the injection site were observed for Pulmo-Guard™ MpB. Finally, 185 animals were subjected to macroscopic lung lesions identification, almost 44% of which showed pathological changes in the lungs and over 34% of them had *M. bovis*-specific lung lesions. Post Mycomune^®^ R vaccination, a significant reduction in the lung lesions identified was observed compared to the control, but it was not reported for *M. bovis*-specific lung lesions. In contrast, there was only a slight reduction in *M. bovis*-specific lung lesions in the group of calves vaccinated with Pulmo-Guard™ MpB, but the number of all lung lesions was higher than in the corresponding control animals. Neither vaccines protected against *M. bovis* upper-respiratory tract colonization and otitis morbidity. Additionally, no significant differences between the vaccinated and control animals in the assessment of both the serum antibody isoclass and selected cytokine responses was shown, however, with visibly increased pro-inflammatory cytokines IL-1β and TNF-α concentrations post vaccination. Ultimately, the efficacy of Mycomune^®^ R and Pulmo-Guard™ MpB vaccines was estimated at 44% and less than 1%, respectively [12]. 

As a result of the impact of *M. bovis* in British cattle herds, the Myco-B One-Dose™ (American Animal Health, Grand Prairie, TX, USA) was imported into the UK under emergency authorization and is now under investigation. The company’s own results claim 10% less deaths and 15% less morbidity in a study of over 500 cattle which were vaccinated no earlier than 60 days of age, though the study has yet to be published [36]. As calves become infected in the first few weeks of life from infected premises it will be interesting to see how this vaccine performs.

### 2.3. Novel Candidates for M. bovis Vaccines

Novel *M. bovis* secreted proteins were identified using proteomic analysis of the *M. bovis* secretome [37]. Of the predicted total of 246 proteins, 60 secreted *M. bovis* proteins were finally selected. Additional analysis identified eight top genes encoding the critical secreted proteins, such as MbovP0038, MbovP0338, MbovP0341, MbovP0520, MbovP0581, MbovP0674, MbovP0693 and MbovP0845, finally considered as virulence-related factors. Six out of eight selected proteins, including MbovP0581, had conserved domains. Additional analysis of these proteins showed that the functions they are involved in, such as multiple cellular activities, chromatic and DNA dynamics, the actual alignment and others, may be important in terms of *M. bovis* virulence and the disease pathogenesis. In the context of a possible vaccine candidate, one secreted protein, MbovP0581, known as ABC transporter ATP-binding protein, appeared to exhibit high immunogenic properties based on its reactivity with sera originated from cattle suffering from *M. bovis* pneumonia [37].

An *M. bovis* virulent strain and one of its attenuated forms previously described in the live *M. bovis* vaccine study were analyzed using proteomic analysis [19,38]. Of the 438 *M. bovis* proteins, 59 were identified as secreted extracellular proteins, most of which, as many as 45, were common to both *M. bovis* strains. Additional analysis showed that 52 of 59 previously identified proteins were classical secretory proteins, the majority of which (40) were shared by both strains. Moreover, 50 out of 59 proteins identified displayed antigenic properties, of which 31 proteins had linear epitopes and were finally selected as the most immunogenic. These proteins had also the affinity of the T-cell epitopes for MHC class I and II molecules. Based on the T-cell epitopes’ affinity for MHC class II molecules, eight proteins were selected with the high affinity and 27 proteins contained the binding affinity for both MHC class I and II molecules, whereas 22 proteins were characterized by a high number of conformational B-cell epitopes. Finally, two secretory proteins, MbovP274 and MbovP570, with strong immunogenic properties were selected and recognized as putative lipoproteins; these are generally considered to be highly antigenic and able to induce both innate and acquired immunity [39]. These proteins were also characterized by high binding affinity of T-cell epitopes for MHC class II molecules. Additional analysis showed that the first belonged to the virulent *M. bovis* strain, while recombinant protein Mbov570 was secreted by both strains. Previous data showed that MbovP274 protein has a highly conserved domain responsible, among others, for virulence and biofilm formation [40]. This protein also contained another conserved domain which is responsible for immunogenic protein transportation across the cell membrane. The recombinant proteins were also able to react with *M. bovis*-positive cattle serum, however, with most seen by rMbov570. The increased expression of some cytokines to the recombinant proteins and their mutants, including immunoregulatory IL-6, IL-12 and IFN-γ, further confirmed the possible use of the two highly immunogenic secreted proteins, MbovP274 and MbovP570, as potential protective antigens and candidates for lipoprotein-based sub-unit vaccine known to induce an extended memory immune response [37,39].

A study carried out on the mechanism of the immune killing of the pathogen identified nine dominant *M. bovis* antigens for specific antibody induction. The results showed that these antibodies are required for *M. bovis* killing by complement, which had a higher killing efficacy than complement alone [41].

## 3. *Mycoplasma mycoides* subsp. *mycoides* Vaccines

### 3.1. CBPP in Sub-Saharan Africa

Data from the World Organization for Animal Health (OIE) show that in 2018, at least 25 countries were affected by CBPP, although more are probably affected as many do not carry out regular or comprehensive surveillance [42]. The countries with the highest prevalence include Ethiopia, Ghana, Tanzania and Angola. Indeed, this has hardly changed over the last 20 years, showing that most attempts at control have been unsuccessful. The disease in sub-Saharan Africa is mostly characterized by a chronic endemicity with occasionally severe outbreaks when naïve herds are exposed to infected animals moved, often illegally, across borders. Moreover, due to the weak economies of many countries, stamping out the movement control, slaughter and compensation seen in Europe are not options [13].

An OIE Scientific Conference in Gaborone in 1994 concluded that vaccination of cattle remained the best way of controlling CBPP, but a vaccine which would confer better immunity than the T1 strain vaccine was urgently needed [43]. Over a quarter of a century later, Jores et al. [44] came to the same conclusions. Calls for studies into the immunology of the diseases have also failed to provide sufficient insight to improve vaccines. No inactivated, sub-unit or attenuated vaccines have been developed which improve upon the live T144 vaccine developed in the 1950s.

The limitations of the T1 strain vaccine have been long recognized: short duration of immunity and tendency to cause adverse reactions, and because it is only semi-attenuated, it could lead, though infrequently, to outbreaks in closed herds [45]. However, mass vaccination had been highly successful in many countries but had failed in others due mainly to the inability to maintain annual vaccination [14]. Nevertheless, this vaccine was sufficient to bring about a huge reduction in cases in many parts of Africa in the 1970s and 1980s. At this time, most national veterinary services were functioning effectively and annual vaccine coverage in some countries was close to 80%, which is the minimum considered necessary to bring about eradication [45]. Today, there are few countries or regions in Africa which are approaching even half this vaccine coverage [46]. Disappointingly, EU-financed initiatives such as the Pan African Rinderpest Campaign, which also covered CBPP, failed to deliver on blanket vaccination and movement control of cattle for the planned five years. Ironically, the eradication of rinderpest had an adverse effect on CBPP control as vaccination with the bivalent rinderpest/CBPP vaccine was discontinued [44].

It is worth reviewing the qualities of the T1/144 vaccine that made it effective, as these can often be forgotten in the haste to develop more speculative and expensive vaccines. Despite various attempts to compromise the potency of the vaccine over the years—developing a bivalent product with rinderpest, producing a streptomycin-resistant vaccine and a deleterious reconstitution procedure [2]—the OIE concluded that the vaccine can effectively protect herds when vaccinations are performed annually [47]; evidence has also been presented that regular vaccination can significantly reduce adverse effects in cattle [48]. Moreover, March [49] showed that simple and inexpensive changes to the current vaccine, such as the use of HEPES buffer systems and the inclusion of pH indicators together with restrictions in the use of 1 M MgSO4 as a vaccine diluent, can increase vaccine yields 10-fold and stability several 100-fold, producing a vaccine which should improve its effectiveness in the field.

### 3.2. Next Generation Vaccines

Therefore, for next generation vaccines to replace T1/44, they should, ideally, be stable, given in a single dose, provide a longer duration of immunity and higher levels of protection and not cause adverse reactions. As March [49] stated prophetically in 2004: “this is by no means assured”. Indeed, since then no vaccines to date have met all these criteria. Despite encouraging immune responses, cattle, given an immune-stimulating complex (ISCOM) vaccine, had similar gross pathological and histopathological scores as non-vaccinated controls [50]. This work provided further evidence that CBPP pathology may be principally due to the action of the host immune system, thus greatly complicating the search for a protective vaccine [49].

A succession of inactivated vaccines has been tried and tested over the last two decades. Two new approaches to vaccination against CBPP were reported by Nicholas et al. [51]. The first consisted of a whole cell mycoplasma vaccine inactivated with saponin—an approach which had previously been found to be protective for bovine mycoplasmosis, contagious agalactia and contagious caprine pleuropneumonia [2]—and the second comprised a recombinant sub-unit vaccine prepared from the highly immunogenic lipoprotein LppQ. In spite of two vaccinations at 6-weekly intervals and high antibody responses there was no evidence in the animals used of any protection afforded by either preparation; indeed, there appeared to be an exacerbation of pathology in the vaccinated animals compared to unvaccinated contact controls. Lesions and fibrin were most extensive and pleural fluid more abundant in vaccinated animals. In the LppQ group, half the cattle died before the end of the experiment, while a quarter died in the saponin group, compared to just under half that died in the control group.

From evidence to date, it would seem that only live vaccines can achieve the levels of protection needed to aid eradication. The multi-cultured V5 strain used in Australia was shown to be at least 97% protective, though severe reactions such as tail loss were seen in 1% of vaccinates [16]. However, this was seen as acceptable and, coupled with movement control and abattoir surveillance, CBPP was eradicated from Australia after over a century of infection.

Very little was known about CBPP in China in the West until the publication of an English language paper called “History of the prevalence and control of CBPP in China” in 2011 [52]. The disease caused great economic losses to China’s cattle industry between the 1950s and 1970s, severely affecting Mao’s “Great Leap Forward”. However, following an eradication campaign initially involving mass vaccination using a novel live, though highly impractical, vaccine, followed by quarantine and slaughter, CBPP was eradicated in 1989 [52]. The vaccine, called Ben-1, was prepared initially in rabbits then passed through sheep to collect larger quantities of pleural fluid for the vaccine; it was reported to have high immunogenicity and protection rates of 100% even after 2 years in China [49]. Recently, this strain was subcultured in mycoplasma medium rather than in sheep for ethical reasons and trialed in Africa. The vaccine was reported to be as effective as T1/44 [44], though the full data has not yet been published.

Using more traditional approaches, Mwirigi et al. [53] compared the protective capacity of the live T1/44 vaccine with two inactivated preparations of the Afadé strain of *M. m. mycoides*, challenged with a virulent strain. The protection levels were poor for the formalin-inactivated vaccine but the heat-inactivated preparation was marginally better than T1/44 at just over 80 and 74%, respectively. These findings indicate that low doses of heat-inactivated *M. m. mycoides* can offer protection at a level similar to the current live-attenuated T1/44 vaccine formulation, though it would be more expensive to produce.

Antibody levels as detected by current serological tests have always been poor indicators of protection and indeed have frequently been linked to post-challenge pathological reactions. Schieck et al. [48] found that animals with CBPP sequestra had significantly higher antibody levels against surface proteins than the animals that cleared mycoplasma from their lungs. The authors suggested that high antibody titers might play a role in the establishment of pathological changes, such as vasculitis. The N terminus of the transmembrane lipoprotein Q (LppQ-N′) of *M. m. mycoides* has been identified as a major antigen and a possible virulence factor in CBPP. Mulongo et al. [54] immunized cattle with purified recombinant LppQ-N′ formulated in Freund’s adjuvant, which showed a strong seroconversion to the lipoprotein. However, the vaccine provoked severe post-challenge glomerulonephritis, probably brought about by the development of antigen–antibody immune complexes. A study by Carozza et al. [55] showed the potential of a viral-vectored prototypic vaccine which gave rapid and strong humoral and cell-mediated immune responses in mice against lipoprotein A, a major antigen of *M. m. mycoides*. This represented a first step in developing a recombinant vaccine against CBPP but is a long way from producing one for use in the field against CBPP. 

A Canadian–Kenyan consortium used reverse vaccinology technology to identify 66 potential vaccine candidates based on the presence of specific antibodies in sera from CBPP-positive animals [56]. Cocktails of five antigens were used to immunize cattle followed by a challenge with a virulent strain. Two of the groups immunized showed protection after challenge and in one group mycoplasma was not recovered from lung specimens. A third group were also negative for mycoplasma and showed a reduced number of animals with lesions. While immunization with some of the antigens conferred protection, others increased immune-related pathology. Experiments are still underway to show whether this vaccine is more protective than T1. However, the group have shown that some of these antigens are able to differentiate vaccinated from infected cattle in an indirect enzyme-linked immunosorbent assay [57].

Mass vaccination, even with a moderate efficacy and duration of immunity vaccine (such as the current T1/44 live vaccine), alone is unlikely to eliminate CBPP according to an epidemiological model for CBPP transmission in pastoral herds of East Africa [45]. Furthermore, vaccine-derived immunity of at least 18 months is required to eliminate CBPP from individual herds. A study in Namibia supported the concept that strategic and targeted antimicrobial treatment can play a critical part in the control, alongside regular and comprehensive vaccination [46].

## 4. The Future

The successful development of protective *M. bovis* vaccines is still a long way off and much research is still needed in this area, especially on developing an animal challenge model. Data on the present commercial vaccines in use today are modest at best, with one showing an efficacy of 1%. Clearly, improvements need to be made before control of this fast-emerging disease is possible. What is clear, however, is that any *M. bovis* vaccine needs to be part of a wider vaccination program involving other respiratory pathogens, including BVD, PI3V, *Mannheimia*, *Pasteurella* and possibly others. Hopefully, the use of bioinformatics tools will allow the proteomics analysis of the *M. bovis* secretome and consequently the detection of novel secreted proteins that can be used not only as diagnostic biomarkers, but also in the development of a potent vaccine for effective control of *M. bovis* infections.

With little immediate prospect of an improved vaccine, the CBPP community does what it has done many times and produces a report providing recommendations for better vaccines [44]. While many of the proposals in the report have been recorded before, the group has also identified development of a robust challenge model as a research priority, as adult cattle are expensive, raise ethical issues and are variable in their response to *M. m. mycoides*, making experiments unreproducible. However, apart from the use of highly speculative tissue explants, there are few other surprises. It is encouraging, however, that the Global Alliance for Livestock Veterinary Medicine (GALVmed) has written: *work is now proceeding to improve the performance and production processes of the existing vaccine* [58]. While this will certainly help, it is annual vaccination with high coverage that remains the key to successful CBPP eradication.

## Figures and Tables

**Table 1:** Reported experimental vaccines against diseases caused by *M. bovis*.

Vaccine Type	Strain or Isolate Used/Source	Adjuvant Used	Inactivator Used	Vaccination Route/Number of Doses/Time Intervals	Components	Age of Vaccinated Animals/Sector	Efficacy	References
inactivated vaccine against pneumonia	strain *M. bovis* KP795974/mastitic cow	saponin and Emulsigen^®^	saponin	sc/one	whole cell	5–6-week-old calves/N/A	yes	[8]
inactivated vaccine against pneumonia	strain *M. bovis* 86B/96/pneumonia case	saponin	saponin	sc/one	whole cell	3–4-week-old calves/N/A	yes	[18]
live vaccine against *M. bovis* disease	strains *M. bovis* P150 or P180 attenuated by multiple in vitro passages of the *M. bovis* strain HB0801	no information	N/A	via the nasal cavity/one	whole cell	5–6-month-old calves/N/A	yes	[19]
quadrivalent vaccine against respiratory disease	respiratory syncytial virus, parainfluenza virus type 3, *M. bovis*, *M. dispar*	Quil A	formalin	sc/two/ 3 weeks intervals	whole cell	3, 5 and 13-week-old calves/beef	partial	[30]
		oil-based				3, 7 and 12-week-old calves/beef		[29]
sub-unit vaccine against *M. bovis* pneumonia and polyarthritis syndrome in cattle	isolates *M. bovis*: Mb1/polyarthritis case and Mb160/chronic pneumonia and polyarthritis syndrome	Montanide ISA 61VG and curdlan	N/A	sc/one	*M. bovis* membrane fractions, protein extracts, recombinant proteins: PdhA, Tuf, PepA, LppB, 0256, OppA, DeoB, P81, and PepQ	6–8-month-old calves/beef	no	[20]
sub-unit vaccine against *M. bovis* pneumonia and polyarthritis syndrome in cattle		Emulsigen^®^, poly I:C and IDR peptide 1002			*M. bovis* membrane fractions, protein extracts, recombinant proteins: PdhA, PepA, Tuf, P48, P81, OppA, LppB, PepQ, 0256 and DeoB		no	[23]
sub-unit vaccine against pneumonia and polyarthritis syndrome	isolates *M. bovis*: Mb1/polyarthritis case and Mb160/chronic pneumonia and polyarthritis syndrome	CpG ODN 2007 and Emulsigen^®^	N/A	sc/two/22 days interval	protein extracts and membrane fractions	5–8-month-old cattle/beef	no	[22]
					membrane fractions			
sub-unit vaccine against pneumonia	*M. bovis*	Quil A	N/A	sc/three/4 days intervals	hydrophobic membrane protein fraction	3-week-old calves/N/A	no	[31]
					a mixture of four purified antigens—Vsp A and non-variable			
inactivated vaccine against *M. bovis* mastitis	strain *M. bovis* California 201	Freud’s complete adjuvant	formalin	sc/three times (with adjuvant) and two times (without adjuvant) intramammary infusion in two quarters/2 weeks intervals	protein	pregnant cows/dairy/N/A	partial	[28]
inactivated vaccine against *M. bovis* arthritis	strain *M. bovis* 1067/mastitic cow	aluminium hydroxyde and Quail A	formalin	im/two/4 weeks interval	whole cell	4–5-week-old calves/N/A	no	[26]
live vaccine against *M. bovis* arthritis	strain *M. bovis* 427/calf joint	no information	N/A	sc or ip/three/10-days intervals	whole cell	1–4.5-month-old calves/N/A	partial (sc) no (ip)	[27]
inactivated vaccine against *M. bovis* arthritis		no information	formalin	sc/three/10 days intervals			no	

Abbreviations: sc: subcutaneous; id: intradermal; ip: intraperitoneally; im: intramusculary; N/A—not applicable.

**Table 2:** Commercial vaccines against diseases caused by *M. bovis*.

Vaccine Type	Strain or Isolate Used/Source	Adjuvant Used	Inactivator Used	Vaccination Route/ Number of Doses/Time Intervals	Country in Which Vaccine is Available	Age of Vaccinated Animals/Sector	Commercial Name
inactivated vaccine against respiratory disease caused by *M. bovis*	two *M. bovis* field isolates and their soluble antigens	proprietary	proprietary	sc/two/14–28 days interval	USA	45 days of age or older calves/N/A	MpB Guard™
inactivated vaccine against respiratory disease and arthritis caused by *M. bovis*	three *M. bovis* field isolates and their soluble antigens	proprietary	proprietary	sc/one	USA	60 days of age or older calves/beef	Myco-B ONE DOSE™

Abbreviations: sc: subcutaneous; N/A—not applicable.

## Data Availability

The data presented in this study are available on request from the corresponding author.
